# Psychoeducation for Fibromyalgia Syndrome: A Systematic Review of Emotional, Clinical and Functional Related-Outcomes

**DOI:** 10.3390/bs13050415

**Published:** 2023-05-15

**Authors:** Carmen M. Galvez-Sánchez, Casandra I. Montoro

**Affiliations:** Department of Psychology, University of Jaén, 23071 Jaén, Spain

**Keywords:** Fibromyalgia Syndrome, psychoeducation, emotional symptoms, clinical symptoms, pain intensity, functional status

## Abstract

Fibromyalgia Syndrome (FMS) is a chronic condition of widespread pain accompanied by several symptoms such as stiffness, fatigue, sleep problems, depression, anxiety, and cognitive deficits. To date, there is no specific treatment for FMS. The European League Against Rheumatism, and the majority of the international recommendations for managing FMS, has claimed psychoeducational intervention as the first step in FMS treatment for adequate symptoms management. However, scientific studies in this regard are scarce, diverse, and with contradictory findings. Results integration from analogous studies could provide a clear presentation of the real clinical value of psychoeducation in FMS. Therefore, the current systematic review aims at exploring the effect of psychoeducation on emotional, clinical, and functional symptoms of FMS patients and encourages researchers towards psychoeducation’s procedure optimization and systematization. The systematic review was conducted according to the guidelines of the Cochrane Collaboration and PRISMA statements. The selected articles were evaluated using the Cochrane risk of bias (ROB) assessment tool. The selected articles were extracted from PubMed, Scopus, and Web of Science databases. The literature search identified 11 studies eligible for the systematic review. The ROB evaluation revealed that 2 of the 11 studies showed a low quality, the other 2 had a moderate quality, and the remaining 7 studies exhibited a high quality. Results showed that psychoeducation is generally included as an important first therapeutic step in multicomponent treatments for FMS. Moreover, psychoeducation generally seems to be quite beneficial in reducing emotional (i.e., number of days feeling emotionally well, general anxiety, depression levels, etc.) and clinical symptoms (levels of fatigue, morning stiffness, pain intensity, etc.), as well as increasing functional status (i.e., general physical function, morning fatigue, stiffness, etc.). Despite that psychoeducation´s clinical benefits are highlighted, there is scarce amount of research on psychoeducation beyond its usefulness as part of multicomponent treatments.

## 1. Introduction

Fibromyalgia Syndrome (FMS) is conceptualized as a chronic condition of widespread pain accompanied by fatigue, sleep problems, depression, and anxiety [1,2,3]. In addition, there is also evidence of cognitive problems in FMS (also called “fibro fog” [4,5]), comprising deficits in attention, perception, concentration, memory, and higher cognitive functions [4,6,7,8,9]. Its prevalence is estimated in 2–4% of the general population, being more frequent in women than in men [2]. In this detail, recent research showed a possible gender bias that seems to lead professionals to underestimate FMS prevalence in men and overestimate it in women [8,9]. FMS negatively impacts the health-related quality of life, especially in activities of daily life, work, career, parenting, interpersonal relationships, and mental health [10,11,12,13].

Related to the negative impact of FMS on the patients’ lives, psychosocial factors (e.g., stress level, social support, personality traits, etc.) seem to play a relevant mediating role between the disease symptoms and their influence on general health and daily activities [14,15]. Moreover, some studies have revealed FMS patients are characterized to a greater extent by personality traits such as alexithymia [16,17], neuroticism [18,19,20], psychoticism [19], avoidant personality [21,22], and type D personality [23]. The last is defined by a high negative affect and social inhibition [24]. FMS patients also tend to exhibit lower self-esteem [6,25,26], difficulties in pain-related self-efficacy [27,28], a negative self-image [29,30], higher levels of pain catastrophizing [19,31], and altered emotional processing [32,33,34,35,36].

The etiology of FMS is still unknown, being a biopsychosocial and multifactorial disorder, in which numerous factors such as genetic predisposition, endocrine factors, stressful life events, physical trauma, sleep problems, and emotional and cognitive disturbances among others, interactively intervene [15]. Unfortunately, given the multitude of factors implicated, no objective clinical test to confirm its diagnosis is currently available [37,38,39,40].

There exist different hypotheses related to FMS etiology. The most empirically supported is the presence of a Central Sensitization to pain phenomenon and deficiencies in endogenous pain and inhibitory mechanisms [41,42,43,44]. These deficiencies are corroborated by greater activation of the “pain neuromatrix” under experimental evocation of pain [45] and abnormally low levels of the neurotransmitters involved in descending pain inhibition (e.g., serotonin or norepinephrine) in FMS [46]. Likewise, inadequate functioning of the inhibitory pathways mediated by the activity of endogenous opioids [47] and an increase in neurotransmitters involved in nociceptive facilitation such as Substance P [48] have been confirmed in FMS. In addition, Central Sensitization to pain in FMS is assumed to prevent the inhibition normally exerted by Aβ fibers responsible for conducting sensory information [49] on the Aδ and C nociceptive pathways [50].

In brief, Central Sensitization to pain in FMS implies hyperexcitability and excessive synaptic efficacy in Central Nervous System (CNS) neurons involved in sensory and nociceptive processing [51,52]. This derives in hyperexcitability of afferent (ascending) nociceptive pathways [53] and inhibition of the efferent (descending) anti-nociceptive pathways [47,54]; which ends up altering the adequate and protective pain signal inhibition [55].

On the behavioral level, the Central Sensitization to pain in FMS is further supported by lower pain thresholds and tolerance as well as the presence of hyperalgesia and allodynia [7,41].

Other hypotheses have discussed the genetic and neurological influence on FMS. Apropos of the first, the HLA class I and II antigen (e.g., DR4) and polymorphisms related to the reuptake of 5-hydroxytryptamine (or serotonin, 5-HTT) such as 5-HTTLPR, or those associated with the catecholaminergic and serotonergic systems such as catechol-*O*-methyltransferase [56,57] have been contemplated. In reference to the second, less density or peripheral neuropathy in small fibers in FMS patients has been most recently discovered [58,59].

Given the etiological mechanisms of FMS are not totally well-known, there is currently no consensus about its appropriate therapy, and sometimes, treatment effects have been claimed to be unsatisfactory [60]. Several interventions, especially the combination of pharmacological and non-pharmacological treatments, however, seem to be useful in lessening FMS symptoms and their impact on the general quality of life [61,62,63,64]. Positive effects of interventions such as cognitive-behavioral therapy (CBT) [65,66,67], mindfulness training [68,69,70], acceptance and commitment therapy (ACC) [71,72], and moderate exercise [65] have been also stated.

Among the mentioned therapies, CBT has been proven to be the most effective therapy for FMS [66,73,74,75]. CBT is composed by psychoeducation, attitudes´ acquisition, training on pain-healthy beliefs, strategies for the maintenance of adequate lifestyles and the prevention of relapses, physiological deactivation techniques (abdominal breathing and relaxation), and cognitive intervention on maladaptive/negative disease beliefs, expectations, and behaviors [74,75,76]. For cognitive intervention, cognitive restructuring, problem-solving, and attention management techniques are used [66,67,75,76]. Though less effective, mindfulness therapy is intended to bolster FMS patients’ ability to be focused on the present moment through meditation, conscious breathing, body scan, and mindful movements [68,69,70]. ACC therapy attempts to increase psychological flexibility, pain acceptance, and treatment process commitment in FMS [77]. Psychological flexibility allows FMS patients to accept and manage a variety of unavoidable events associated with pain, instead of investing energy in fighting with them. ACC reduces avoidance behaviors, facilitates acceptance and contact with the present, and promotes a state of mental calm [71,72,78].

On the subject of moderate exercise interventions, FMS patients are advised to avoid a sedentary lifestyle and keep active at the physical and social levels [62]. The Body Mass Index (BMI), social isolation, general functioning, and well-being are demonstrated to be improved and controlled by exercise and physical activity interventions [60,70,79,80]. Obesity seems to be a common problem in FMS patients which is associated with greater pain sensitivity, poorer sleep quality, reduced physical strength and flexibility [81,82], and marked reduction of cognitive performance [82].

Most therapy approaches directed to FMS patients include psychoeducation as the first phase of the treatment due to its relevance in patients’ adherence and prognosis [66,67]. Indeed, according to the European League Against Rheumatism (EULAR) recommendations for managing FMS, psychoeducational intervention has to be the first mandatory step in FMS patients’ treatment [83]. In line with the above, Multidisciplinary Pain Education Programs (MPEP) have been observed to be significantly beneficial in Central Pain Sensitization (CPS) conditions [84,85]. FMS is well-known to be the prototypical CPS condition [86].

Broadly, the main purposes of educative interventions are: (1) to give patients and caregivers the necessary information about the pathologies´ characteristics and treatment options; and (2) to provide details on potential positive effects on family functioning and patient behavior [87]. Patient´s education may be defined as any set of educative activities planned by qualified professionals and aimed at providing information and/or restructuring the patients´ disease perception and, therefore, improving patients’ health-related behaviors and/or status [88].

FMS´s psychoeducation generally involves information about the distinction between acute and chronic pain, FMS nature, disease contributing factors, treatments that are most safe and effective, and the symptoms´ characteristics; and coping strategies, among others [83,89,90,91]. Moreover, educative programs may contribute to increase therapeutic adherence as previously reported, self-confidence, self-esteem, and pain self-efficacy in FMS patients [92]. An efficient psychoeducation in FMS patients might positively impact the disease’s treatment and prognosis [92].

As a whole, patient education, as a part of a wider multidisciplinary program, might be not only useful but crucial for FMS patients’ symptom management [67,89]. Psychoeducation in FMS patients may increase the knowledge and understanding of the disease, being a therapeutic strategy itself that positively impacts the rest of the treatment [62,93,94]. To the extent that patients understand what FMS is and deconstruct the myths surrounding this disease: (1) a better active and healthy facing of the disease will be achieved [95]; (2) FMS patients will be more likely to draw upon support networks and socio-health resources [95]; and (3) to develop a more favorable attitude towards the disease, which will also have a potential impact on the disease treatment and prognosis [93,94,95].

Nonetheless, further research is needed to determine a framework from which to develop non-pharmacological interventions (i.e., psychological therapies) guidelines in FMS [15], including the optimization and systematization of psychoeducational programs. Scientific studies in this regard are scarce, methodologically diverse (i.e., different combinations of therapies, several protocols of psychoeducation, variability related to the number of sessions of psychoeducation, etc.), and with findings in different directions [83,86]. Results integration from analogous studies could provide a clear presentation of the real clinical value of psychoeducation in FMS.

Based on the previous aforementioned literature, the current systematic review intended to: (1) explore the effect of psychoeducation on emotional (e.g., depression and anxiety), clinical (e.g., pain), and functional (e.g., fatigue, health-related quality of life and impact´s disease) FMS related-outcomes; and (2) encourage further research on clinical settings psychoeducation methodization.

## 2. Materials and Methods

### 2.1. Search Strategy

This systematic review was performed according to the guidelines of the Cochrane Collaboration and reported according to the Preferred Reporting Items for Systematic Reviews and Meta-Analyses (PRISMA) [96]. The search terms were as follows: “fibromyalgia” and “psychoeducation”. The terms were extracted from the Medical Subject Headings (MeSH). The PICO question was: what is the effect of psychoeducation on emotional, clinical, and functional related outcomes in FMS?

**Figure 1:**
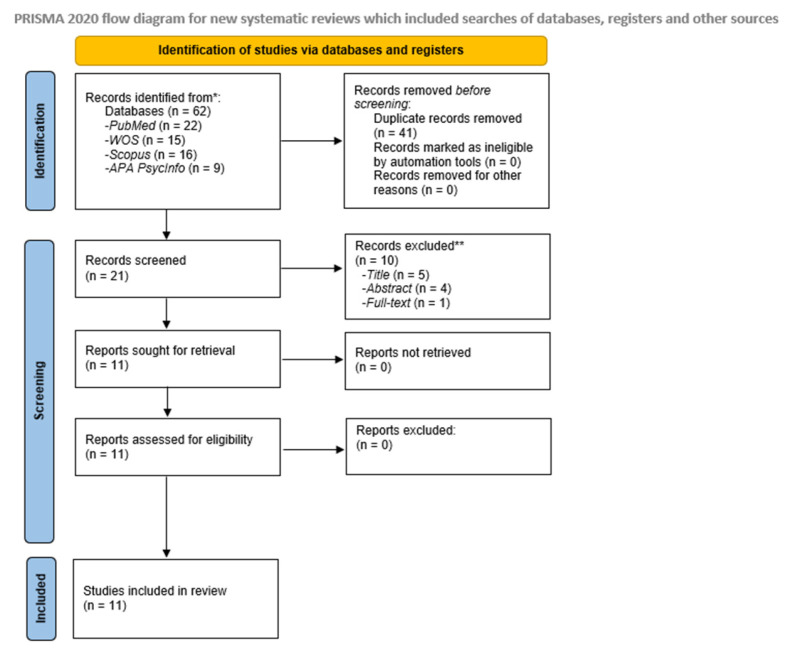
Flow diagram for relevant eligible studies related to psychoeducation effects on emotional, clinical, and functional Fibromyalgia Syndrome related-outcomes.

The selected articles were extracted from PubMed, Scopus, and Web of Science databases. All articles were screened; those that fulfilled the inclusion criteria for full-text analysis were selected. Among these, the titles and abstracts were revised to remove those not relevant for the review. Afterward, the resulting articles were screened in depth for eligibility. To attain a final set of articles to be reviewed, the full texts of relevant articles were retrieved and screened based on the inclusion and exclusion criteria. The PRISMA flowchart (Figure 1) shows the screening and selection process for the inclusion of studies. The last search was conducted on 1 February 2023.

### 2.2. Eligibility Criteria

Inclusion criteria were as follows: (1) peer-reviewed original studies of FMS and psychoeducation (i.e., longitudinal studies, pilot studies, pilot randomized controlled trials, randomized controlled clinical, quasi-experimental replicated single-case/small group designs, and uncontrolled and controlled pre-post-test studies); (2) studies comprising adult patients (≥18 years old) with an official diagnosis of FMS; and (3) studies written in English. The exclusion criteria were: (1) review article or meta-analysis; (2) comment, editorial, case report, letter, or meeting/congress abstract; and (3) non-English publication.

### 2.3. Data Extraction and Quality Assessment

The study characteristics, methodologies, and results were extracted independently by C.M.G.-S. and C.I.M. in the following sequence: first author, study name, country, year of publication, study design, sample size and the number of participants in each study group, participant age and sex, and the diagnostic criteria of FMS. The study characteristics are presented in Table 1.

With the objective of assessing the quality of the selected articles, the risk of bias (ROB) in each study was evaluated by C.M.G.-S. and C.I.M. according to the Cochrane ROB assessment tool. This tool comprises seven items evaluating ROB: random sequence generation (selection bias), allocation concealment (selection bias), blinding of participants and personnel (performance bias), blinding of outcome assessment (detection bias), incomplete outcome data (attrition bias), selective reporting (reporting bias), and other bias. For each item, the ROB was graded as high, medium, or low. Moreover, the global quality of each article was also assessed.

### 2.4. Data Synthesis

In view of the aim of the current systematic review, the authors checked each study´s main objectives, the methodology, and if there were or were not control groups included. In addition, the characteristics and purposes of FMS psychoeducational programs (i.e., the effect on emotional, clinical, and/or functional symptoms, the content of sessions, the number of interventions, etc.) were analyzed together with the pre-post and follow-up results. The clinical relevance of the main findings and the principal limitations of each research were also determined (see Table 1 for more detail). In addition, the specific subject-matter of the FMS psychoeducational programs are detailed in Table 2. Finally, the biases of each study were analyzed and reported in the Section 3.4 and Table 3 The latter analyses were performed to determine the effect of psychoeducation in FMS with the commitment to improve FMS clinical intervention and guide future research lines in this field.

## 3. Results

### 3.1. Literature Search and Study Characteristics

From among a total of 62 articles identified by database searches, 21 were finally selected for screening after removing duplicates. A general PRISMA flow chart was devised detailing the number of studies excluded at each stage of the screening (Figure 1). An analysis of 11 full-text articles was conducted in order to determine their eligibility for the present review. Only 11 articles fulfilled the inclusion criteria; therefore, they were subjected to the data extraction (Table 1) and quality assessment (Table 3) processes. The selected studies were published between 2002 and 2022. Of the 11 studies, 1 was a Delphi technique [97], 2 were pilot studies [98,99], and 8 were randomized controlled trials [100,101,102,103,104,105,106,107]. Four studies were conducted in Spain [98,100,101,102], 2 in Sweden [99,107], 2 in Canada [104,105], 1 in The United States of America [106], 1 in Ireland, the United Kingdom, North America, and other non-specified countries [103], and 1 in Brazil [97]. The 11 selected studies included a total of 1659 participants (age range: 27–60 years old), of which 1073 were FMS patients.

**Table 1:** Characteristics of relevant eligible studies related to psychoeducation effects on emotional, clinical, and functional Fibromyalgia Syndrome´ related-outcomes.

First Author (Publication Year), Study Name, Country	Objectives	Study Design/Procedure	Sample Size [Mean ± Age (SD)]	FMS Diagnostic Criteria	Instruments and Variables	Results
Antunes et al. (2022). Amigos de Fibro (Fibro Friends): Validation of an Educational Program to Promote Health in Fibromyalgia. Brazil. [97].	To validate a multidisciplinary educational health promotion program for individuals with FMS.	Delphi technique. Procedure: Phases: (1) Development of Amigos de Fibro; (2) Content validation of Amigos de Fibro; (3) Adjusting the Amigos de Fibro; (4) Final assessment of Amigos de Fibro; and (5) Final version of Amigos de Fibro.	N = 23 health professionals (expert judges). 10 males (43.5%) and 13 females (56.5%). Aged between 31 and 40 years old (39.2%). N = 45 individuals with FMS (target audience). 4 males (9%) and 41 females (91%). Aged between 31 and 40 years old (38%).	2016 ACR, revised version.	Groups of professionals and individuals with FMS listed their demands through the focus group. Evaluation of Amigos de Fibro, built with the information and results obtained from the first round, regarding the objectives, proposed themes and initiatives, relevance, writing style, and structure of the program (with specialists and individuals with FMS). Final evaluation of the material after the corrections are made, based on the judges’ suggestions.	Content validity index (CVI) ≤ 0.78 and coefficient kappa ≤ 0.61. All 25 items evaluated in both groups presented considerable minimum CVI by CVI and the kappa coefficient. Global CVI of Amigos de Fibro, by the specialist judges, was 0.90; and 0.95 by the target audience judges. The kappa coefficient of the expert judges was 0.90 and that of the target audience judges was 0.85. Amigos de Fibro was considered with adequate content validity and internal consistency.
Pérez-Aranda et al. (2021). Do humor styles predict clinical response to the MINDSET (MINDfulneSs & EducaTion) program? A pilot study in patients with fibromyalgia. Spain. [98].	To explore the role of humor styles in predicting clinical changes after the multicomponent intervention (MINDSET) that combines mindfulness and psychoeducation for FMS patients.	Pilot Study. * Procedure: MINDSET intervention: 4 psychoeducation sessions about FMS, based on a previously validated program, and 4 sessions of mindfulness training, based on the Mindfulness-Based Stress Reduction curriculum. Psychoeducation Sessions: 2 h, twice per week, run by health psychologists in a group setting of 8–10 patients. Intervention added on to the patient’s usual care (i.e., medication). No additional active treatments.	N = 35 FMS patients. N = 34 (97.1%) FMS female patients [54.97 ± 8.65].	1990 ACR.	FFMQ-15. FIQR. HSQ. PGIC.	FMS patients: affiliative humor and positive/negative ratio humor styles had a unique predictive effect on self-reported clinical changes. Association between humor styles with functional impact and mindfulness facets. Some humor styles may imply a better disposition in patients to learn and implement the concepts and resources that the intervention offered.
Melin et al. (2018). Psychoeducation against depression, anxiety, alexithymia, and fibromyalgia: a pilot study in primary care for patients on sick leave. Sweden. [99].	(1) To try the feasibility of ASSA in a Swedish primary care setting; (2) to explore associations between symptoms of depression, anxiety, alexithymia, and MUPS.	Pilot Study. * ¤ Procedure: ASSA began with 8 group sessions—‘the Affect School’, which were followed directly by 10 individual sessions—‘the Script Analysis’. All 27 respondents one-week post-intervention terminated ASSA within 20 weeks from the start. Script Analysis sessions were performed with one instructor, either the physiotherapist, the GP, or one social counselor. Affect School comprised 8 weekly, 2-h sessions, of a 5–7 participant group, led by the same instructors (one psychotherapist, one physiotherapist, and one GP) during all sessions. Psychoeducation Sessions: 8 weekly 2-h sessions with a 5–7 participant group led by two instructors followed by 10 individual hour-long sessions. Follow-up: 18 months.	N = 36 patients. 29 female patients (81%). Median age 39, range 27–60 years. (N FMS patients: 2 [6%]).	Not specified.	TAS-20. SASB. SCI-93. EuroQol ‘health barometer’ (100 mm—VAS).	Patients: one-week post-intervention median test score changes were significantly favorable for 9 of 11 measures (depression, anxiety, alexithymia, MUPS, general health, self-affirmation, self-love, self-blame, and self-hate); at 18 months post-intervention the results remained significantly favorable for 15 respondents for 7 of 11 measures (depression, alexithymia, MUPS, general health, self-affirmation, self-love, and self-hate).
Feliu-Soler et al. (2016). Cost-utility and biological underpinnings of Mindfulness-Based Stress Reduction (MBSR) versus a psychoeducational program (FibroQoL) for fibromyalgia: a 12-month randomized controlled trial (EUDAIMON study). Spain [102].	(1) To examine the effectiveness and cost-utility for FMS patients of MBSR as an add-on to treatment as usual (TAU) versus TAU + the psychoeducational program FibroQoL, and versus TAU only; (2) to examine pre-post differences in brain structure and function, as well as levels of specific inflammatory markers in the three study arms; and (3) to analyze the role of some psychological variables as mediators of 12-month clinical outcomes.	12-month randomized controlled trial. * ¤ Procedure: Protocol in progress. Psychoeducation Sessions (FibroQoL): 8, 2-h sessions. Three treatment arms: (1) TAU + MBSR; (2) TAU + FibroQoL; (3) TAU. Control Group: TAU (pharmacologic treatment + counselling about aerobic exercise adjusted to patients’ physical limitations). Active control group: TAU + FibroQoL. FibroQoL: a psycho-educational program for FMS patients based on a consensus document drawn up by the Health Department of Catalonia. Planned follow-up: 12 months.	N = 180 FMS female patients. N = 60 FMS female patients per group.	1990 ACR.	Sociodemographic-clinical questionnaire. Structured Clinical Interview for DSM Axis I Disorders (SCID-I). **Screening measures:** MMSE. **Primary Outcome:** FIQR. **Secondary Outcomes:** CSRI. EQ-5D-5L. FFMQ. FSDC. HADS. MISCI. PCS. PIPS. PSS. SCS. **Other measures:** CEQ. PGIC. PSIC. Log of out-session for MBSR and psychotherapeutic practices. Adverse events of the interventions. Neuroimaging MRI. Brain structure: VBM. Inflammatory markers: Blood samples.	Protocol in progress.
Bourgault et al. (2015). Multicomponent interdisciplinary group intervention for self-management of fibromyalgia: a mixed-methods randomized controlled trial. Canada [104].	To evaluate, quantitatively and qualitatively, the efficacy of the PASSAGE Program—a multicomponent interdisciplinary group intervention for the self-management of FMS.	A mixed-methods randomized controlled trial. * ¤ Intervention (INT) vs. waitlist (WL). Qualitative group interviews with a subset of patients were also conducted. Procedure: Intervention: PASSAGE Program (a structured multicomponent interdisciplinary group intervention aimed at reducing FMS symptoms and maintaining optimal function through the use of self-management strategies and patient education). 9 group sessions with 8 participants lasting 2.5 h each. Each session involved 3 major components: (1) psycho-educational tools; (2) CBT-related techniques; and (3) patient-tailored exercise activities. Follow-up: 3 months.	N = 28 INT Group. 26 females (92.9%). [49.98 ± 9.23]. N = 28 WL Group. 26 females (92.9%). [46.74 ± 11.42].	1990 ACR.	**Primary outcomes:** Change in pain intensity (0–10). **Secondary outcomes:** Fibromyalgia severity. Pain interference. Sleep quality. Pain coping strategies. Depression. Health-related quality of life. PGIC. Perceived pain relief.	FMS patients: the intervention had a statistically significant impact on the three PGIC measures. At the end of the PASSAGE Program, the percentages of patients who reported pain relief and perceived overall improvement on their pain levels, functioning, and quality of life were significantly higher in the INT Group than in the WL Group. The same differences were observed 3 months post-intervention. The results of the qualitative analysis were in line with the quantitative findings regarding the efficacy of the intervention. The improvement, however, was not reflected in the primary and secondary outcomes.
Dowd et al. (2015). Comparison of an Online Mindfulness-based Cognitive Therapy Intervention With Online Pain Management Psychoeducation: A Randomized Controlled Study. Ireland, the UK, North America, and other countries [103].	To test the effectiveness of a computerized mindfulness-based cognitive therapy intervention (MIA) compared to computerized pain management psychoeducation (PE) in a randomized study.	A randomized controlled study. * ¤ Procedure: Participants in each condition received 12 sessions of treatment, twice per week for 6 weeks. MIA intervention was based on established mindfulness meditation and emotional regulation programs shown to be effective for chronic pain. Psychoeducation Sessions: based on many of the common elements found within pain management programs. The PE program was presented in a series of twice-weekly emails containing written information about chronic pain self-management. Follow-up: 6 months.	N = 124 chronic pain patients. 112 females (90.3%) and 12 males). [44.53 ± 12.25]. N MIA group = 62 participants (N FMS patients: 15). N PE group = 62 participants. (N FMS patients: 18).	Not specified.	**Primary Outcomes:** Pain interference (BPI). Psychological Distress (HADS). **Secondary Outcomes:** Pain Intensity: 2 NRS from BPI. PCS. SWL. Average Pain Pain Right Now. CPAQ. MAAS. PGIC.	FMS patients: both groups showed improvements in pain interference, pain acceptance, and catastrophizing from pre-treatment to post-treatment and at follow-up. Reduced average pain intensity from baseline to post-treatment for both groups, but not at follow-up. Increases in subjective well-being, were more pronounced in the MIA than in the PE group. MIA group: greater reduction in pain ‘right now’, and increases in their ability to manage emotions, manage stress and enjoy pleasant events on completion of the intervention.
Luciano et al. (2013). Cost-Utility of a Psychoeducational Intervention in Fibromyalgia Patients Compared With Usual Care. An Economic Evaluation Alongside a 12-Month Randomized Controlled Trial. Spain. [101].	(1) To determine the effectiveness of adding psychoeducational treatment implemented in general practice to usual care for patients with fibromyalgia; (2) to analyze the cost-utility of the intervention from health care and societal perspectives.	12-month randomized controlled trial. * ¤ Procedure: See Luciano et al., 2011.	See Luciano et al., 2011.	1990 ACR.	See Luciano et al., 2011.	FMS patients who received psychoeducation: greater improvement on global functional status, physical functioning, pain, morning fatigue, stiffness, and depression. It was confirmed the long-term clinical effectiveness of a psychoeducational treatment program for FMS implemented at the primary care level and the cost-utility from a healthcare and societal perspective.
Luciano et al. (2011). Effectiveness of a Psychoeducational Treatment Program Implemented in General Practice for Fibromyalgia Patients. A Randomized Controlled Trial. Spain. [100].	To examine whether a psychoeducational intervention implemented in primary care is more effective than usual care for improving the functional status of patients with FMS.	Randomized Controlled Trial. * ¤ Procedure: The treatment program is based on a consensus document developed by an expert panel in 2005 and published in 2006 by the Catalan Health Department. Psychoeducation Sessions: 9, 2-h sessions (5 sessions of education and 4 sessions of autogenic relaxation), delivered over a 2-month period (1-afternoon session per week), run by GP and rheumatologist, with a maximum of 18 patients per group. Six separate intervention groups were performed. Intervention group: Usual care from their GP + psychoeducational program. Control group: Usual care from their GP. Usual care from their GP: pharmacologic treatment + counselling about aerobic exercise adjusted to patients’ physical limitations. Follow-up: 12 months.	N = 211 participants. N = 105 intervention group. FMS female patients (97.2%) [55.17 ± 8.58]. N = 106 control group. FMS female patients (98.1%) [55.42 ± 8.63].	1990 ACR.	Sociodemographic Questionnaire. Chronic Medical Conditions Checklist. Marlowe-Crowne Social Desirability Scale. FIQ. STAI.	FMS patients who received psychoeducation: a 2-month psychoeducational intervention improves the functional status to a greater extent than usual care, at least in the short-term. The social desirability bias did not explain the reported outcomes. Trait anxiety was associated with response to treatment.
Mannerkorpi et al. (2009). Pool exercise for patients with fibromyalgia or chronic widespread pain: a randomized controlled trial and subgroup analyses. Sweden [107].	To evaluate the effects of pool exercise in patients with fibromyalgia and chronic widespread pain and to determine characteristics influencing the effects of treatment.	Randomized controlled trial. * ¤ Procedure: 20-session exercise programme combined with a standardized 6-session education programme based on self-efficacy principles with an active control group, which undertook the same education programme. Psychoeducation Sessions: The education programme, which was designed to introduce strategies to cope with FMS symptoms, consisted of 6 1-h sessions, conducted once a week for 6 weeks. The programme was led by a physiotherapist. The pedagogical approach was based on the active participation of the patients through discussions and practical exercises. The control group received the same education programme. Exercise programme: comprised 20 sessions of 45-min pool exercise once a week for 20 weeks in temperate (33 °C) water, supervised by a physiotherapist. The exercise was planned to permit individual progress, aiming to improve overall function and to motivate regular physical activity. Follow-up: 11–12 months after the baseline.	N = 166: 134 FMS female patients + 32 chronic widespread pain (CWP) female patients. N = 81 Exercise—Education Group. [44.60 ± 9.26]. Intervention group. N = 85 Education Group. [46.50 ± 8.30]. Control Group.	1990 ACR.	**Primary outcomes:** FIQ total score. Body functions (6MWT). **Secondary outcomes:** Pain (the FIQ Pain). Fatigue (the FIQ Fatigue). Depression (HADS-D). Health-related quality of life (SF36). Amount of leisure time physical activity (LTPAI). **Exploratory outcomes:** Clinical manifestations of stress (SCI). Multiple dimensions of fatigue (MFI-20). Experience in physical activity (ITT and PP). Note: PP is defined as attendance at least 60% of the sessions.	FMS patients: The exercise-education programme showed significant, but small, improvement on health status in patients with fibromyalgia and chronic widespread pain, compared with education only. Patients with milder symptoms improved most with this treatment.
Rook et al. (2007). Group Exercise, Education, and Combination Self-management in Women With Fibromyalgia. United States [106].	To evaluate and directly compare the effects of 4 common self-management interventions on well-established measures of functional status, symptom severity, and self-efficacy in women with fibromyalgia.	Randomized Controlled Trial. * ¤ Procedure: Both exercise programs involved approximately 60 min of activity per session. Each session began with a brief warm-up of walking on a treadmill at a comfortable pace and then progressed to a self-determined level of moderate effort for a predetermined amount of time. All participants, regardless of fitness level, began with 5 min of walking and increased a maximum of 2 to 4 min weekly following a predetermined progression. The AE group progressed to a total of 45 min of walking. The ST group reached a maximum of 20 min of treadmill walking followed by 25 min of strength training movements. Psychoeducation Sessions: The Fibromyalgia Self-Help Course (FSHC) is a 7-session program that teaches individuals with fibromyalgia about the condition and self-management skills. Sessions were 120 min long every 2 weeks. All FSHC instructors were certified by the Arthritis Foundation. Follow-up: 6 months.	N = 207 enrolled and randomized FMS female patients. N = 138 FMS female patients who completed the intervention. N = 35 Aerobic and flexibility exercise (AE) Group [48.00 ± 11.00]. N = 35 strength training, aerobic, and flexibility exercise (ST) Group. [50.00 ± 11.00]. N = 27 the Arthritis Foundation’s Fibromyalgia Self-Help Course (FSHC) Group. [51.00 ± 12.00]. N = 38 a combination of ST and FSHC (ST-FSHC) Group. [50.00 ± 11.00].	1990 ACR.	**Primary outcomes:** Change in physical function from baseline to completion of the intervention (FIQ and SF-36). **Secondary outcomes:** Social and emotional function, symptoms (FIQ, the bodily pain and vitality subscales of the SF-36, and BDI). Self-efficacy (adapted Arthritis Self-Efficacy Scale).	FMS patients: progressive walking, simple strength training movements, and stretching activities improve functional status, key symptoms, and self-efficacy in women with fibromyalgia actively being treated with medication. The benefits of exercise are enhanced when combined with targeted self-management education. Appropriate exercise and patient education be included in the treatment of fibromyalgia.
King et al. (2002). The effects of exercise and education, individually or combined, in women with fibromyalgia. Canada [105].	To examine the effectiveness of a supervised aerobic exercise program, a self-management education program, and the combination of exercise and education for women with fibromyalgia (FMS).	Randomized controlled trial with repeated measures design. * ¤ Procedure: The intervention programs were based upon principles of self-management (Bandura’s social cognitive theory). Treatment programs ran simultaneously for 12 weeks. Due to the large number of subjects required, the programs were offered on 5 different occasions over a 2 year period (winter–spring once, fall–winter, and spring–summer twice each). Education Group: met once a week for one and a half to 2 h per session. Exercise and education group: combined exercise and education programs. The educational component was the same as for the education-only group. The exercise-only group met twice per week and on the third day met for education and then exercise. Control group: On the day of the initial assessment, they were given a page of instructions for basic stretches and 5 items related to general coping strategies. They were contacted once or twice throughout the 12-week period to ensure they were filling out their logbook and to answer any questions. Subjects from the control group were offered one of the intervention programs at the end of the follow-up period. Follow-up: 3 months.	N = 170 FMS female patients. N = 46 Exercise Group. [45.2 ± 9.4]. N = 48 Education Group. [44.9 ± 10.0]. N = 37 Exercise & Education Group. [47.4 ± 9.0]. N = 39 Control Group. [47.3 ± 7.3].	1990 ACR.	SE. SE Pain. SE Function. SE Coping with symptoms. FIQ. 6MW. Tender Point Count. Total Survey Site Score.	FMS patients: subjects receiving the combination of exercise and education and who complied with the treatment protocol improved their perceived ability to cope with other symptoms. A supervised exercise program increased walking distance at post-test, an increase that was maintained at follow-up in the exercise-only group. Results demonstrate the challenges with conducting exercise and education studies in persons with FMS.

**Note:** * Pre and Post Evaluation. ¤ Follow-up assessment. **Abbreviations:** 6MW: Six Minute Walk; 6MWT: 6-min walk test; ACR: American College of Rheumatology’s Criteria; ASSA: Affect School and Script Analyses; BPI: Brief Pain Inventory; CBT: Cognitive-Behavioral Therapy; CEQ: Adapted version of the Credibility/Expectancy questionnaire; CPAQ: Chronic Pain Acceptance Questionnaire; CSRI: Client Service Receipt Inventory; CVI: Content Validity Index; EQ-5D-5L: EuroQoL-5D questionnaire; FFMQ: Five Facet Mindfulness Questionnaire; FFMQ-15: Five-Facets Mindfulness Questionnaire; FIQ: Fibromyalgia Impact Questionnaire; FIQR: Revised Fibromyalgia Impact Questionnaire; FMS: Fibromyalgia Syndrome; FSDC: Fibromyalgia Survey Diagnostic Criteria; FSHC: Fibromyalgia Self-Help Course; GP: General Practitioners; HADS: Hospital Anxiety and Depression Scale; HSQ: Humor Styles Questionnaire; INT: Intervention; ITT: Intention-to-treat; LTPAI: The Leisure Time Physical Activity Instrument; MAAS: Mindful Attention Awareness Scale; MBSR: Mindfulness-Based Stress Reduction; MFI-20: The Multidimensional Fatigue Inventory; MIA: Mindfulness-based Cognitive Therapy Intervention; MIR: Magnetic Resonance Imaging; MISCI: Multidimensional Inventory of Subjective Cognitive Impairment; MMSE: Mini-Mental State Examination; MUPS: Medically Unexplained Physical Symptoms; NRS: numerical rating scales; PCS: Pain Catastrophizing Scale; PE: Pain Management Psychoeducation; PGIC: Patient Global Impression of Change; PIPS: Psychological inflexibility in pain scale; PP: per-protocol; PSIC: Pain Specific Impression of Change; PSS: Perceived Stress Scale; SASB: Structural Analysis of Social Behavior assessment tool; SCI: The Stress and Crisis Inventory; SCI-93: Stress and Crisis Inventory-93; SCS-12: Self-Compassion Scale-short form; SE: Chronic Pain Self-Efficacy Scale; STAI: State Trait Anxiety Inventory; SWL: Satisfaction with Life Scale; TAS-20: Toronto Alexithymia Scale-20 items; TAU: Treatment as Usual; UK: United Kingdom; VAS: Visual Analogue Scale; VBM: Voxel-Based Morphometry; WL: Waiting List.

### 3.2. Psychoeducation and Emotional, Clinical, and Functional Related-Outcomes in Fibromyalgia Syndrome

Psychoeducation has generally been included as part of the multi-component treatments for FMS patients and the success of these treatments has in fact been attributed to the characteristic combination of therapeutic strategies [98,102,103,104,105,106,107].

The first group of researchers proposed a multi-component program, namely the MINDSET (MINDfulneSs & EducaTion) program [98], which combined mindfulness and psychoeducation. In the reference study, Pérez-Aranda et al. [98] found that affiliative humor and positive/negative ratio humor styles had a unique predictive effect on self-reported clinical changes [98]. Additionally, significant correlations between humor styles and functional impact and mindfulness facets were also reported [98]. The authors concluded that some humor styles may imply a better disposition in patients to learn and implement the concepts and resources offered by mindfulness and psychoeducation sessions [98]. Participants showed a notable degree of adherence (74% of attendance to the sessions) and considered the intervention satisfactory (9/10), useful (8.9/10), recommendable (8.7/10), and non-aversive (0.5/10), in a post-treatment ad-hoc opinion survey [98].

Other researchers also combined psychoeducation with mindfulness-based therapies. For instance, Feliu-Soler et al. [102] created a protocol to evaluate the cost-utility and biological underpinnings of a Mindfulness-Based Stress Reduction (MBSR) intervention versus a psychoeducational program (FibroQoL) for FMS. The protocol proposed a 12-month randomized controlled trial (EUDAIMON study) with the purpose of: (1) analyzing the aforementioned cost-utility; (2) examining pre-post differences in brain structure and function; (3) determining the level of specific inflammatory markers in the three study arms or branches; and (4) exploring the mediational role of psychological variables on the 12-month clinical outcomes [102]. Unfortunately, the protocol remains to be tested.

Similarly, Dowd et al. [103] compared an Online Mindfulness-based Cognitive Therapy Intervention (MIA) with an Online Pain Management Psychoeducation (PE), in a randomized controlled study. Dowd et al. [103] reported that both groups (i.e., PE and MIA groups) showed improvements on pain interference, pain acceptance, and catastrophizing from pre-treatment to post-treatment and follow-up. Average pain intensity was also reduced from baseline to post-treatment, but not at follow-up, for both groups [103]. Increases in subjective well-being were more pronounced in the MIA than in the PE group [103]. Upon completion of the intervention, MIA group showed a greater reduction in pain ‘right now’, and an increase in the ability to manage both emotions and stress and enjoy pleasant events [103].

By contrast, Luciano et al. [100] conducted a randomized control trial for testing a multi-component treatment combining usual care and psychoeducation. The psychoeducational intervention consisted in a 2-month program that was proved to improve at short-term the functional status to a greater extent than the usual care [100]. Social desirability bias—also evaluated in Luciano et al. [100]—did not explain the reported outcomes, and trait anxiety was associated with response to treatment [100]. The 12-month follow-up showed a greater improvement on the global functional status, physical functioning, pain, morning fatigue, stiffness, and depression in FMS patients who did receive psychoeducation [101]. The sensitivity analysis suggested that the intervention was cost-effective even after imputing all missing data [101]. As the authors advised, its implementation at primary care will benefit 3 FMS patients rather than 1, as befall with the standard treatment [101].

Multi-component programs combining psychoeducation and physical exercises were also found in three of the reviewed articles [104,105,106,107]. In the study of Bourgault et al. [104] the beneficial effects of a multicomponent interdisciplinary group intervention for the self-management of FMS, named the PASSAGE program, were explored. The PASSAGE program was aimed at reducing FMS symptoms and maintaining optimal function through the use of self-management strategies and patient education. This program included 3 key intervention components: (1) psycho-educational tools; (2) CBT-related techniques; and (3) patient-tailored physical exercise activities. The percentages of patients reporting pain relief and perceiving an overall improvement on pain levels, functioning, and quality of life were significantly higher in the INT Group (Intervention Group) than in the WL Group (Waiting list Group) [104].

King et al. [105] studied the effects of physical exercise (walking exercises) and education, individually or combined, in FMS patients. The authors observed that FMS patients receiving the combination of physical exercise and education, and complied with the treatment protocol, significantly improved their perceived ability to cope with other symptoms.

Likewise, Mannerkorpi et al. [107] implemented an intervention of pool exercise and education for patients with FMS and chronic widespread pain and compared it with single education. Authors observed that the pool exercise-education program showed significant—but lesser—improvement on health status in FMS and chronic widespread pain patients, compared with single education.

Rook et al. [106] combined physical exercise, education, and self-management in an intervention program addressed to FMS patients. Results pointed out that progressive walking, simple strength training movements, and stretching activities enhanced functional status, the characteristics symptoms of the disorder, and self-efficacy in FMS patients [106]. Moreover, the physical exercise´s benefits were further intensified when was united with targeted self-management education [106].

Despite the aforementioned results, the reviewed studies showed scarce research on psychoeducation benefits beyond its usefulness as part of multicomponent treatments. Moreover, the differences in the protocol and the variability between interventions were also prevalent. Solely Melin et al. [99] conducted a pilot study to test the effectiveness of a psychoeducation program for depression, anxiety, and alexithymia in FMS and chronic pain patients. Nine of the 11 factors evaluated were significative favorable one-week post-intervention. This was the case of depression, anxiety, alexithymia, Medically Unexplained Physical Symptoms (MUPS), general health, self-affirmation, self-love, self-blame, and self-hate. Eighteen months post-intervention, changes remained significantly favorable for 7 of the 11 factors (i.e., depression, alexithymia, MUPS, general health, self-affirmation, self-love, and self-hate) but this result was restricted to 15 patients. Unfortunately, only 6% of the sample included in the study of Melin et al. [99] were FMS patients. Findings must therefore be considered with caution.

### 3.3. Structure of the Fibromyalgia Syndrome Psychoeducation Programs

The specific subject-matter of the FMS psychoeducational programs are detailed in Table 2. As a whole, the programs included information related to the diagnosis, typical symptoms (i.e., pain, fatigue, and sleep problems), and manners to cope with them, usual disease progress, comorbid medical conditions, potential etiology, information about: (1) the influence of psychosocial factors on symptoms; (2) treatments (pharmacological and non-pharmacological); (3) FMS typical myths; (4) the benefits of regular physic exercise; (5) the typical barriers to behavior change; and (6) chronic pain´ resources (i.e., personal, derived from the relatives, the social network, communities, and/or social organizations such as patients associations, etc.) and care services, among others [97,98,99,100,101,102,103,104,105,106,107]. The authors agreed on emphasizing the need to provide enough time to solve possible doubts in patients and ended up the program with a summary session [97,98,99,100,101,102,103,104,105,106,107].

It should be not overlooked, the lack of systematization between the different psychoeducation programs analyzed. Reliability and validity analyses of the programs’ structure were also predominantly missed. Only Antunes et al. [97] validated the “Amigos de Fibro (Fibro Friends)” program; an educational program to promote health in FMS. On purpose, Antunes et al. [97] informed of an adequate content validity and internal consistency for the program.

**Table 2:** Structure of the Fibromyalgia Syndrome psychoeducation programs.

Study (Author and Year)	Psychoeducation Program Content
Antunes et al. (2022) [97].	- Program introduction and socializing. - Knowing fibromyalgia. - Health production and care. - Family and work. - Body practices and physical activity. - Adequate and healthy eating. - Health and well-being. - Pharmacological approach. - Integration of the activities. - Integrative and complementary practices. - Integration of the activities. - Occupational performance. - Integration of the activities. - Sleep quality. - Retrospective.
Pérez-Aranda et al. (2021) [98].	- Introduction of the course. Overview of what is known about FMS, diagnosis, causes, symptoms, and treatment. - Myths about FMS and tips for maintaining the pillars of good health: rest, healthy diet, exercise practice, no consumption of tobacco/drugs, moderate intake of caffeine/alcohol, and following doctors’ prescriptions. - Discussion of the most frequent emotions that FMS patients usually cope with. Tips for emotion management and communication skills. - What services and resources do FMS patients have? Health care system, communitarian system, and summary of the course.
Melin et al. (2018). Affect School [99].	- Affect theory presented during the 8 Affect School sessions. - One hand-out is delivered per session. General affect theory is presented during all sessions. Specific affect theory for each innate affect is presented following the scheme below: Sessions: (1) Joy; (2) Fear; (3) Interest and surprise; (4) Shame; (5) Anger; (6) Distaste and dissmell; (7) Distress; (8) Pain. - Script theory displayed at all sessions: (1) Affects and experiences together form the individual scripts; (2) How we act in different situations and how we interpret experiences are depending on our scripts; (3) Scripts are formed by family rules and common cultural rules for how affects should be handled; (4) Intensity and expressions of emotion are controlled by scripts; (5) Affects can be completely suppressed and thereby unconscious. - Coffee break. - Affect discussion. - Main topics for the affect discussions follow the program for the eight sessions. Questions used in the affect discussion are: (1) Tell of a situation you felt the affect… (2) How do you know that you feel…? (3) Do you feel … in a particular place in your body? (4) Does it happen often that you feel…? (5) How do you know that someone else is…? (6) Can you understand and accept another person’s…?
Feliu-Soler et al. (2016). FibroQoL. [102].	- Introduction and general information. Patients’ Expectations. History and epidemiology of FMS. Common symptoms in FMS. Physiological mechanisms involved in the genesis of pain. - Collect information on the goals of each patient, explain differences between physical and emotional pain, clarify differences between hypnosis and self-hypnosis, and administer the hypnotizable test, and hypnosis “safe place”. - Diagnosis and prognosis. Pharmacological and non-pharmacological treatments. The current model of health care in Catalonia and units specialized in the treatment of FMS patients. - Discussion of goals and the difficulties that obstruct them, emphasize common personality characteristics, highlight exceptions to the problem, hypnosis “candle and bubbles”. - Strategies to increase self-esteem and regulate emotions. Pain experience and recurrent invalidation. Social support from family and close friends. - Exploration of possible changes, the difference between acute and chronic pain, hypnosis: “imagination of a journey”. - Reviews the goals, asks for a future possible change (the miracle question), commitment to the consolidation of the changes, hypnosis: “Watch a photo album”. - Benefits of physical exercise in FMS and closing remarks.
Bourgault et al. (2015) [104].	- Introduction. - FMS symptoms. - Exercise and physical activity as part of FMS management. - Psychological tools as part of FMS management. - Energy and capacity management. - The vicious circle of chronic pain. - Pharmacological and non-pharmacological treatment of FMS. - Review and summary.
Dowd et al. (2015) [103].	- What is pain? Acute pain. Challenges of chronic pain: disability and suffering. - Pain concepts that cause misunderstandings. The experience of pain. Pain behavior. Physical injury and damage. - Physiology of pain. Role of the spinal gate. Role of endorphins. - Physical Deconditioning. What is physical deconditioning. Doing too much. - Avoiding physical deconditioning. Living within your limitations. Use good pacing procedures. Use Caution during dangerous times. - Activity pacing. The Importance of planning ahead and pacing yourself. The activity-rest cycle? Benefits of the activity—rest cycle. - Physical fitness. Physical fitness defined. Step to appropriate fitness. - Sleep difficulties. Why sleep is important? Developing healthy sleep habits. - Sleep difficulties continued. Tips for when you can’t sleep at night. - Interacting with your medical doctor. The Importance of having a primary care doctor. Doctor—Patient relationship. Problems with being believed or taken seriously. - Your relationship with your medical doctor. A bill of rights for people with pain. Maintaining reasonable expectations. Communicating with your doctor. - Review.
Luciano et al. (2011) [100]. Luciano et al. (2013) [101].	- Information about typical symptoms, usual course, comorbid medical conditions, potential causes of the illness, the influence of psychosocial factors on pain, current pharmacologic and nonpharmacologic treatments, the benefits of regular exercise, and the typical barriers to behavior change.
Mannerkorpi et al. (2009) [107].	- Symptoms and explanatory theories for long-lasting pain. The session started by listing the patients’ symptoms on a flip chart, followed by a discussion of these symptoms. A short presentation of theories for long-lasting pain was given, followed by a discussion of the participants’ own theories and beliefs. A short relaxation exercise was performed while seated. - Pain and pain alleviation. Physical activity and exercise. A short presentation of the local (gate theory) and central (central nervous system) levels of pain modulation and strategies for pain alleviation was given, followed by a discussion of the participants’ experience. The participants were encouraged to use different techniques, including physical activity and relaxation. A contract for physical activity for the forthcoming week was written. A short relaxation exercise was performed while seated. - Stress, pain, and depression. Feedback for physical activity during the past week was given and a new contract for activity for the forthcoming week was written. A short presentation of theories about stress was given, followed by the participants’ own experience of what makes them stressed and how they prevent and alleviate stress. A short relaxation exercise was performed while seated. - Physical relaxation and body awareness. Feedback for physical activity during the past week was given and a new contract for activity for the forthcoming week was written. Continuation of discussion about stress. Methods for active and passive relaxation and body awareness were presented and practiced. - Lifestyle. Feedback for physical activity during the past week was given and a new contract for activity for the forthcoming week was written. - Identification of possible causes of increases in pain and stress and opportunities to do something about them were discussed. The participants were asked to write down their own plans for changes, according to a model that was presented. - Lifestyle. Feedback for physical activity during the past week was given and a new contract for activity for the forthcoming week was written. Continuation of the topic introduced in session 5.
Rook et al. (2007) [106].	The Fibromyalgia Self-Help Course (FSHC): - A 7-session program that teaches individuals with fibromyalgia about the condition and self-management skills. - Materials promoted basic self-management techniques to accomplish daily activities and manage symptoms and suggested ways to incorporate wellness activities, including exercise into daily life. - Information is provided through a series of lectures (5–15 min) with facilitated group discussion and supplementary readings.
King et al. (2002) [105].	- Information regarding the potential cause of FMS. - Goal setting with respect to a significant goal for the subject. - Maximizing energy for household chores or personal activities. - Pain or fatigue coping strategies. - Benefits of exercise. - Evaluating alternative therapies. - Barriers to behavior change. Notes: (1) Sessions were focused away from pain and other symptoms as much as possible and refocused on leading a well-balanced life. (2) One session included family and/or friends of study participants, mainly to educate participants about FMS and how to assist someone with FMS.

**Abbreviations:** FMS: Fibromyalgia Syndrome.

### 3.4. Risk of Bias

The ROB evaluation (see Table 3) revealed that 2 of the 11 studies showed a low quality [98,99], the other 2 demonstrated a moderate quality [97,103], and the 7 remaining exhibited a high quality [100,101,102,103,104,105,106,107]. The most frequent biases were performance bias (blinding of participants and personnel) and detection bias (blinding of outcome assessment).

The recognized limitations by authors included: (1) the small number of FMS patients taking part in the studies [98,99]; (2) the lack of a control group [98,99] or an active control group [101]; (3) the absence of other clinical, coping-related, and mindfulness measures [98]; (4) the use of insufficient assessment instruments [101]; (5) difficulties in the control and assessment of the home practice [103]; (6) the non-analysis of common comorbid psychiatric disorders (i.e., major depression, personality disorders, etc.) [100], and other relevant variables such as the pharmacological intake [99]; and (7) the non-report of the follow-up assessments [100]. The last did not let prove whether intervention led to neither permanent improvement on FMS patients’ functional status or determine the direct and indirect costs derived from interventions—an aspect especially relevant when policymakers are involved.

Other limitations were also found by the present review, such as: (1) the non-specifications of the number of male and female patients [98,99,103]; (2) the non-sample match in sex, with a high predominance of female [97,98,99,100,101,102,103,104,105,106,107]; (3) the absence of male FMS patients [102]; (4) the non-specification of the primary and secondary outcomes [98,99,100,101,105]; (5) the non-perform of the follow-up assessments [98]; (6) the lack of information about participants´ nationality [103]; (7) the absence of a pilot study testing the program utility and effectivity [97]; (8) the non-specification of the FMS diagnostic criteria used [99,103]; (9) the general low sample size [99]; (10) the non-use of any measure for patients experiences and opinions (i.e., the Patient Global Impression of Change [PGIC]) [98,99,100,101]; (11) the lack of details on the sample characteristics related to the diagnosis [99]; and (12) the non-clarification of how the sample size was calculated [98,99,107]. By contrast, 7 studies a priori determined the sample size [100,101,102,103,104,105,106].

The aforementioned biases and limitations need to be overcome in future studies to better understand the effect of psychoeducation on FMS patients and improve the FMS interventions.

**Table 3:** Risk of Bias Assessment of relevant eligible studies.

Study (Author and Year)	Random Sequence Generation (Selection Bias)	Allocation Concealment (Selection Bias)	Blinding of Participants and Personnel (Performance Bias)	Blinding of Outcome Assessment (Detection Bias)	Incomplete Outcome Data (Attrition Bias)	Selective Reporting (Reporting Bias)	Other Bias	General Assessment (Low, Medium, High)
Antunes et al. (2022) [97].	H	L	H	L	L	L	Yes	Medium
Pérez-Aranda et al. (2021) [98].	H	H	H	H	L	L	Yes	Low
Melin et al. (2018) [99].	H	H	H	H	L	L	Yes	Low
Feliu-Soler et al. (2016) [102].	L	L	L	L	L	L	Yes	High
Bourgault et al. (2015) [104].	L	L	L	L	L	L	Yes	High
Dowd et al. (2015) [103].	L	L	H	H	L	L	Yes	Medium
Luciano et al. (2013) [101].	L	L	L	L	L	L	Yes	High
Luciano et al. (2011) [100].	L	L	L	L	L	L	Yes	High
Mannerkorpi et al. (2009) [107].	L	L	L	L	L	L	Yes	High
Rook et al. (2007) [106].	L	L	L	L	L	L	Yes	High
King et al. (2002) [105].	L	L	L	L	L	L	Yes	High

**Note:** L: Low, M: Medium, H: High.

## 4. Discussion

The present systematic review aimed at exploring the effect of psychoeducation in emotional, clinical, and functional related-outcomes in FMS patients and encouraging further research on clinical settings psychoeducation optimization.

The review confirmed psychoeducation is generally included as a part of other multi-component treatments [98,102,103,104,105,106,107]. Indeed, among the studies reviewed, only Melin et al. [99] evaluated a single psychoeducational program confirming its feasibility for patients on sick leave due to depression and/or anxiety. Psychoeducation was mostly evaluated in interaction with mindfulness [98,102,103], TAU (Treatment as Usual) [100,101] or physical exercise [104,105,106,107]. On behalf of the first, the MINDSET (MINDfulneSs & EducaTion) program [98] demonstrated that some humor styles might lead to higher readiness to learn and use the concepts and resources offered by mindfulness and psychoeducation intervention in FMS patients [98]. Nonetheless, though authors considered MINDSET clinically relevant for the emotions and emotions´ regulation in the treatment of FMS, the specific effect of psychoeducation sessions was not studied at all.

The EUDAIMON study proposed by Feliu-Soler et al. [102] consisted in a protocol of a 12-month randomized controlled trial, to examine the cost-utility and biological underpinnings of Mindfulness-Based Stress Reduction (MBSR) in comparison with a psychoeducational program (FibroQoL) in FMS. However, as in the above study, no firm conclusion about psychoeducation was drawn. The proposed protocol lacked of a real implementation.

Fortunately, the Online combined program MIA-PE by Dowd et al. [103] did evidence equivalent changes across several evaluated outcomes for participants in both conditions (psychoeducation and mindfulness) over time (e.g., improvements on pain interference, pain acceptance, and catastrophizing and reduced average pain intensity). Even so, the MIA intervention showed a number of unique benefits (e.g., greater reduction in pain ‘right now’, increases in the ability to manage both emotions and stress and enjoy pleasant events). Note that the level of participant attrition in the study highlighted the relevance of fostering to a greater extent participant engagement in future online chronic pain programs [103]. In this regard, after the situation provoked by the COVID-19 the online therapies have been postulated as essential. COVID-19 consequences have been especially negative for chronic pain patients in general [108], and FMS patients in particular [108]. The wide accessibility and low-cost of online intervention methods establish their offer as rather fundamental and worthy in FMS patients [109,110,111,112].

With respect to TAU and psychoeducation, Luciano et al. [100] showed a greater increase in patients’ functional status compared to usual care by itself [100]. Specific improvements were reported in physical function, the total number of days of feeling well, levels of pain, general fatigue, morning fatigue, stiffness, anxiety (less trait anxiety), and depression [100]. In the follow-up of the Luciano et al. [101] study, the long-term clinical effectiveness of the psychoeducational treatment was confirmed in FMS patients [101].

Regarding the multi-component programs incorporating psychoeducation and physical exercises [104,105,106,107], particularly, the PASSAGE [104] program aimed at reducing FMS symptoms and maintaining optimal function through the use of self-management strategies and patient education through sessions involved 3 major components: (1) psycho-educational tools; (2) CBT-related techniques; and (3) patient-tailored exercise activities. Authors informed of significantly greater levels of pain relief and perceived overall improvement on functioning, and quality of life in the INT Group compared to the WL Group [104]. King et al. [105] also reported an enriched in the perceived ability to cope with other symptoms in FMS patients who received the combination of exercise and education and follow entirely the treatment protocol. Mannerkorpi et al. [107] pointed out that the implementation of an education and pool exercise combination in FMS and chronic widespread pain patients led to a slight augmentation in the health status of both patients´ groups compared with the single education. Consistently, Rook et al. [106] using a program based on Group Exercise (progressive walking, simple strength training movements, and stretching activities), Education, and Self-management in FMS patients observed that progressive walking, simple strength training movements, and stretching activities improved functional status, key symptoms of the disorder, and self-efficacy in FMS patients [106]. Interestingly, the benefits of physical exercise (i.e., progressive walking, simple strength training movements, and stretching activities) increased when combined with targeted self-management education [106].

The evaluated studies confirm a positive value of education in chronic pain, specifically in FMS [98,102,103,104,105,106,107]; and its benefits on the patient’s functional status [100,101,107], physical function, pain [100,101,104], fatigue, stiffness, depression [100,101], anxiety [99,100,101], and quality of life [104]. Findings are also in line with the scientific evidence suggesting that multicomponent therapies in FMS may reduce pain, fatigue, depressed mood, and health-related quality of life disabilities, as well as, improve self-efficacy, pain coping, and physical fitness, compared to the single education, WL, or TAU [104,105,106,107,113,114,115,116,117].

Hence, the present systematic review confirms psychoeducation as clinically relevant for FMS, but especially when used in combination with other treatments. This entails support for the majority of evidence-based guidelines for the management of FMS that highly recommend multicomponent treatment [83,91,118]. For instance, the Multi-Component Cognitive Behavioral Therapy (MCCBT) for FMS is recommended by Division 12: Society of Clinical Psychology of the American Psychological Association (APA) [73]. The MCCBT for FMS often includes the following components and techniques: (1) education about the syndrome, including its nature and the patient´s role in its care; (2) symptom self-management skills targeting pain, fatigue, sleep, cognition, mood, and functional status (e.g., deep relaxation and breathing, graded activation, pleasant activity scheduling, and sleep hygiene); and (3) lifestyle change promoting skills targeting barriers to change, unhelpful thinking styles, and long term maintenance of change (e.g., stress management, goal setting, structured problem solving, reframing, and communication skills) [73].

Despite the reported benefits, it is important to highlight the scarce studies on psychoeducation beyond its usefulness as part of multicomponent treatments and, e.g., the limited small FMS sample size [99]. Indeed, Melin et al. [99] was the only study evaluating a single psychoeducation program, but unfortunately did not account for a satisfying FMS sample size. Further research lines, especially for FMS patients, on psychoeducation effectiveness have been revealed to be required. Research on FMS male patients, and FMS patients from other countries (apart from Europe)—note sociocultural factors influencing psychoeducation interventions—related to the effectiveness of psychoeducation and the etiology of the disease, and/or validation of psychoeducation are encouraged to enhance evidence-based clinical practice in FMS treatment and create and optimize a unique and common protocol between researchers and clinicians. The latter could allow addressing the cost-effectiveness or cost-utility of nonpharmacological treatments in FMS patients.

## 5. Conclusions

To conclude, to date, psychoeducation has been generally included in other FMS multicomponent treatments. These multicomponent therapies seem to be useful for FMS treatment, with psychoeducation an undoubtedly essential component. However, the reviewed evidence does not certainly support the total utility of psychoeducation as a single treatment. Its beneficial effects are mostly enhanced in a multi-component treatment or in relation to other interventions. In addition, a lack of a psychoeducational systematic and homogeneous protocol between studies is discerned. Though further research is required, the present findings value the importance of ensuring that patients benefit from the enhancer-positive effects of psychoeducation.
